# Long Non-coding RNA and mRNA Expression Change in Spinal Dorsal Horn After Exercise in Neuropathic Pain Rats

**DOI:** 10.3389/fnmol.2022.865310

**Published:** 2022-03-30

**Authors:** Ge Song, Wei-Ming Zhang, Yi-Zu Wang, Jia-Bao Guo, Yi-Li Zheng, Zheng Yang, Xuan Su, Yu-Meng Chen, Qing Xie, Xue-Qiang Wang

**Affiliations:** ^1^Department of Rehabilitation Medicine, Ruijin Hospital, Shanghai Jiao Tong University School of Medicine, Shanghai, China; ^2^The Second Clinical Medical School, Xuzhou Medical University, Xuzhou, China; ^3^Department of Sport Rehabilitation, Shanghai University of Sport, Shanghai, China; ^4^Department of Rehabilitation Medicine, Shanghai Jiao Tong University Affiliated Sixth People’s Hospital, Shanghai, China; ^5^Department of Rehabilitation Medicine, Shanghai Shangti Orthopaedic Hospital, Shanghai, China

**Keywords:** swim, spinal dorsal horn, sequencing, neuropathic pain, lncRNA, mRNA

## Abstract

Exercise can help inhibition of neuropathic pain (NP), but the related mechanism remains being explored. In this research, we performed the effect of swimming exercise on the chronic constriction injury (CCI) rats. Compared with CCI group, the mechanical withdrawal threshold of rats in the CCI-Swim group significantly increased on the 21st and 28th day after CCI surgery. Second-generation RNA-sequencing technology was employed to investigate the transcriptomes of spinal dorsal horns in the Sham, CCI, and CCI-Swim groups. On the 28th day post-operation, 306 intersecting long non-coding RNAs (lncRNAs) and 173 intersecting mRNAs were observed between the CCI vs Sham group and CCI-Swim vs CCI groups. Then, the biological functions of lncRNAs and mRNAs in the spinal dorsal horn of CCI rats were then analyzed. Taking the results together, this study could provide a novel perspective for the treatment for NP.

## Introduction

Neuropathic pain (NP) is a primary lesion or disease of the somatosensory system, the symptoms and signs of which include spontaneous pain, allodynia, hyperalgesia, and paresthesia (Jensen and Finnerup, 2014; Colloca et al., 2017; Cavalli et al., 2019). Long-term pain not only reduces the sleep quality of NP patients, but also leads to decreased quality of life and psychological state (Colloca et al., 2017; Wu et al., 2019). Epidemiological surveys show that the prevalence of NP is 7–10% (van Hecke et al., 2014). Global aging has rendered NP a major public health issue and socioeconomic burden (Colloca et al., 2017; Bouhassira, 2019). Previous studies have found that the majority of available NP treatments have mild effects or dose-limiting side effects, and many NP patients have pain that cannot be properly treated (Attal and Bouhassira, 2015; Cooper et al., 2016; Gierthmühlen and Baron, 2016). Therefore, exploring safe and effective treatments for NP is necessary.

Exercise is widely used in the medical field as a therapeutic method and as a new approach to relieve various painful conditions (Dobson et al., 2014; Cooper et al., 2016; Kami et al., 2017; Palandi et al., 2020; Zhao et al., 2020; Zhou et al., 2020; Zheng et al., 2021; Peng et al., 2022; Wu et al., 2022). Previous studies have reported that swimming and treadmill running could significantly improve mechanical allodynia, cold allodynia, and heat hyperalgesia, while suppress the level of inflammatory cytokines in animal NP models (Cooper et al., 2016; Kami et al., 2017). Swimming, as an effective method of reducing pain in rats, is an attractive form of exercise for patients with NP. Swimming can relieve the load on the aching limbs and coordination problems affected by pain in most patients, especially in the elderly (Cooper et al., 2016; Kami et al., 2017). However, the exact mechanism by which swimming alleviates NP is insufficiently understood and requires further exploration.

Recent developments in RNA-sequencing (RNA-seq) technology have enabled the screening of differentially expressed genes (DEGs) in the NP process and improved the understanding of the mechanism of NP (St John Smith, 2018). Long non-coding RNAs (lncRNAs), as non-protein coding RNAs with more than 200 nucleotides in length, have gene regulation functions. Earlier research established the participation of lncRNAs in the pathological process of NP by modulating pain-associated genes and altering neuronal excitability (Zhou et al., 2017a,b; Wu et al., 2019). Published studies also found that the spinal dorsal horn is the site of greatest concern in basic researches on NP. The spinal dorsal horn is the first station for the central nervous system to receive pain afferent signals (Cohen and Mao, 2014; Tsuda, 2016). After preliminary integration, the information is uploaded to the thalamus, and then the information is further transmitted to the cerebral cortex, thus causing pain. The dorsal horn of spinal cord is an important node for upward transmission of pain signals (Guo and Hu, 2014). However, no study evaluating the function of exercise in spinal dorsal horn transcriptomes in an NP animal model has yet been published. Therefore, in this research, we demonstrate the effect of swimming on the transcriptome of chronic constriction injury (CCI) rats, and utilized RNA-seq technology analyzing DEGs with their biological function. The genetic changes induced by exercise afford potential intervention targets for the development of NP.

## Materials and Methods

### Animals and Exercise Training

The SLAC Laboratory (Shanghai, China) afforded us with Sprague Dawley rats. The rats were all 6-week-old males weighing between 180 and 200 g. The rats were given standard water and rat chow. Their ambient temperature was controlled at 24 ± 1°C and the light and dark cycle was 12/12 h. All experimental processes were authorized by the Ethics Committee of Scientific Research of Shanghai University of Sport.

**FIGURE 1:**
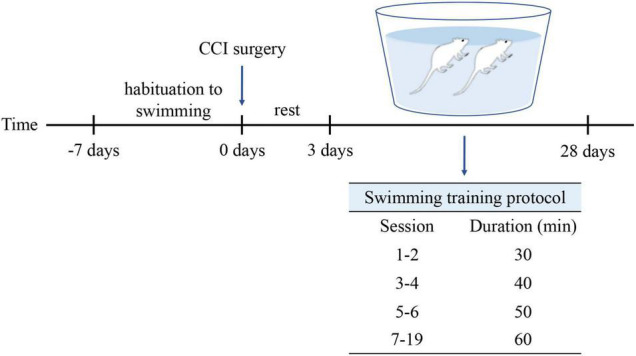
Protocol for swimming exercise. Swimming was included 1-week habituation before CCI surgery and 4-week formal training after CCI surgery.

The rats (*n* = 18) were grouped randomly into three groups: the Sham group (*n* = 6), the CCI group (*n* = 6), and the CCI-Swim group (*n* = 6). Adaptive feeding was conducted for 1 week. The rats in Sham and CCI groups were routinely fed and did not participate in swimming exercise, while rats in the CCI-Swim group were adapted to swim for 1 week preoperatively. Swimming time gradually increased from 10 min on day 1 to 60 min on day 6. On the third day after the CCI operation, rats in the CCI-Swim group were made to swim for a total of 19 sessions, and the swimming time was gradually increased. The first and second sessions involved swimming for 30 min each day, the third and fourth sessions involved swimming for 40 min each day, and the fifth and sixth sessions involved swimming for 50 min each day. In the seventh to ninth sessions, the rats swam for 60 min each time (Figure 1). This swimming training program is based on a previously published animal swimming exercise program that was improved by our research group. The rats were made to swim in a plastic box (82 cm × 60 cm × 59 cm) at a temperature of 35–37°C. After swimming, the rats are caught and dried with a heat blower (Almeida et al., 2015).

### Chronic Constriction Injury Models

We performed CCI on the sciatic nerve of the rats on the basis of a previous study (Jaggi et al., 2011). First, we anesthetized the animal with 5% isoflurane. Then, the skin of the rats’ right lower limb was cut, and the muscle and connective tissue were bluntly dissected to locate the sciatic nerve. Four chromic catguts (4.0 silk) were ligated on the sciatic nerve at an interval of approximately 1 mm. The sciatic nerve of rats in the Sham group was exposed but not ligated. The skin was then sewn with 5.0 silk sutures, and the rats were allowed a recovery period after the operation.

### Mechanical Withdrawal Threshold

We performed mechanical withdrawal threshold (MWT) tests before and after CCI at 3, 7, 14, 21, and 28 days. The rats were placed in transparent glass boxes for 20 min to acclimatize before each behavioral test. The MWT test was performed by harmless stimulation to the posterior plantar of the rats. Von Frey wires (4–180 g, Aesthesio, Danmic Global, United States) were applied to the right hind paw, and the intensity of the Von Frey wires increased from small to large until the rats exhibited the desired behavior. Each rat was tested at least three times, at 5 min intervals.

### Histological Examination

On the 28th day after CCI operation, three rats in each group (i.e., Sham, CCI, and CCI-Swim groups) were killed, and the spinal cord was collected for histopathological analysis. The spinal cord was fixed in 4% paraformaldehyde solution at 4°C for 90 min. After phosphate-buffered saline (PBS) cleaning for 15 min, the samples were then respectively soaked in 15 and 30% sucrose solution for 24 and 48 h. The tissue samples were placed in compound compounds (SAKURA, 4583), sliced (14 μm thick), and dyed with hematoxylin and eosin (HE). The slices were rinsed with PBS for 5 min, soaked in hematoxylin staining solution (Servicebio, G1005-1) for 4 min, and then washed under running water for 20 min. After washing, 1% hydrochloric acid solution was used for differentiation for 3 s. Rinsing was repeated for 15 min. The slides were kept in 0.1% eosin staining solution (Servicebio, G1005-2) for 3 min and then soaked in 85, 95, and 100% alcohol solutions for 1 min in sequence. After soaking in xylene I and xylene II for 1 min, the tissue samples were sealed with neutral resin and then observed under an Olympus BX53 microscope (Tokyo, Japan).

### Sample Collection and RNA-Sequencing

On the 28th day after surgery, the remaining rats (*n* = 9, three in each group) were anesthetized with 3% pentobarbital sodium and instilled with saline (250 mL, 4°C) via the aorta. The corresponding positions of L4–L6 were located on the surgical side of the spine, and the spinal canal was opened to remove the L4–L6 spinal dorsal horn. TRIzol reagent was utilized to extract total RNA from the spinal dorsal horn. RNA quantification and qualification were conducted prior to sequencing. We established sequencing libraries through NEBNext^®^ Ultra™ RNA Library Prep Kit for Illumina^®^ (NEB, United States) and appended the index code to attribute sequence of each sample. Then, the PCR products were purified by AMPure XP system, and the library quality was evaluated on Agilent Bioanalyzer 2100 system. Finally, the library preparation was sequenced by Illumina Hiseq platform.

### Differentially Expressed Genes and Bioinformatics Analysis

The DESeq2 R package (1.10.1) was utilized to analyze the identified DEGs. DEG selection criteria is *P*-values < 0.05 and | log2 Fold Change (FC) | > 1.

Gene Ontology (GO) analysis was executed by the clusterProfiler R package. The KEGG database allows for genome deciphering, which enables a better understanding and analysis of the interactions, reactions, and relational networks of biomolecules produced by sequencing experimental techniques.^1^ The ClusterProfiler R package was utilized to detect DEGs enrichment in KEGG pathways. The significant enrichment criterion for GO analysis and KEGG pathway analyses was *P* < 0.05.

### Validation by Quantitative Real-Time PCR

Quantitative real-time PCR (qRT-PCR) was used to detect the DEGs to verify the accuracy of the sequencing results. Total RNA was reverse transcribed into cDNA by Evo M-MLV RT Kit with gDNA Clean for qPCR II (Cat. No. AG11711). The qRT-PCR was executed by SYBR^®^ Green Premix Pro Taq HS qPCR Kit II (Cat. No. AG11702) and run in a real-time system (Thermo Fisher Scientific, CA, United States). U6 and β-actin were identified as internal controls for lncRNAs and mRNAs, respectively, and the 2^–ΔΔ*Ct*^ approach was applied to describe relative expression ratios. The specific primer sequences in this work are listed Table 1.

**TABLE 1:** The primers utilized in qRT-PCR.

Primer	Forward	Reverse
XLOC_274480	AGATACCAGTCATCCT ACCTGC	CGAGACATCGTATCATG GAGTCA
XLOC_105980	CGGTCTCCAGCTAGTTA TCCAC	GGCAACCGGAGAAAGAA TTGAAC
XLOC_134372	AGTGAAATACATTGCTGT CCTGTC	CACTGGGAGGATATGG TAAGGCA
AABR07047899.1	TGATCCAAAAGCTGC CCGACA	GAACAGAATCTCCTTGC GACA
C3	TCTTGTCACTGCCC ATCACT	GAGTCCTTCACAT CCACCCA
Sgk1	CTCAGTAACAAAACCC GAGGC	ATTCCCGGTCAAGAA ATGCTC
Dnah7	ATGATACCCGTTCTG CACCA	TGACGAGGGTGCT TCTGAAA
Vwa3a	TCCACGAGAAGGCA ATGGTA	TATCTGCACAGCTT TCCCGA
U6	CTCGCTTCG GCAGCACA	AACGCTTCACGAA TTTGCGT
β-Actin	TGTCACCAACTGG GACGATA	GGGGTGTTGAAGGT CTCAAA

### Statistics

Two-way repeated-measures analysis of variance with Tukey’s *post hoc* analysis was conducted to evaluate the results of weight measurement and behavioral tests. The results of qRT-PCR were determined by independent-sample *t*-tests. All of the data were analyzed by Graphpad Prism 9.0 and presented as mean ± standard error of the mean (SEM). The standard of statistical significance was *P* < 0.05.

## Results

### Chronic Constriction Injury Model Identification and Mechanical Withdrawal Threshold Analysis

**FIGURE 2:**
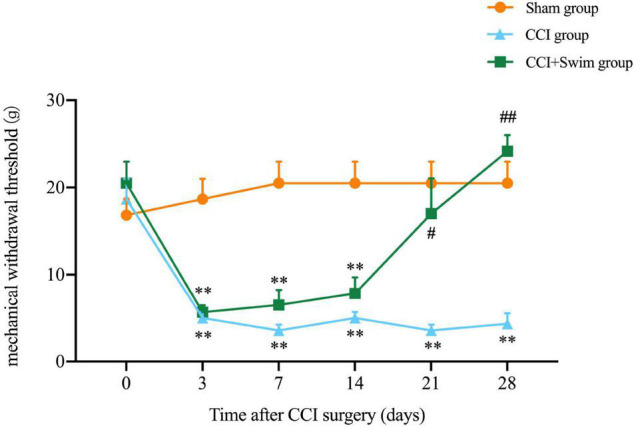
Effect of exercise therapy on mechanical allodynia induced by chronic constrictive injury (CCI). Analyses was adopted by two-way repeated measures analysis of variance (ANOVA) with Tukey’s *post hoc* analysis. The average is denoted by mean ± standard error of mean (SEM), *N* = 6 per group. ^**^p < 0.01, for comparisons of the Sham group vs the CCI group or CCI-Swim group. ^#^*p* < 0.05, ^##^*p* < 0.01 for comparisons of the CCI-Swim group vs the CCI group.

In this study, the NP model of rats involved CCI on the right hind limb. MWT test was performed in all three groups before and after operation (Figure 2). No significant difference in body weight among the three groups. Contrast to rats in the Sham group, animals in CCI group showed higher mechanical allodynia sensitivity from day 3 to 28 after CCI surgery; the CCI-Swim group also exhibited higher MWT on days 3, 7, and 14 after surgery. MWT was significantly improved in the CCI-Swim group on the day 21 and 28 after operation in contrast to the CCI group.

### Histological Examination of the Spinal Dorsal Horn

**FIGURE 3:**
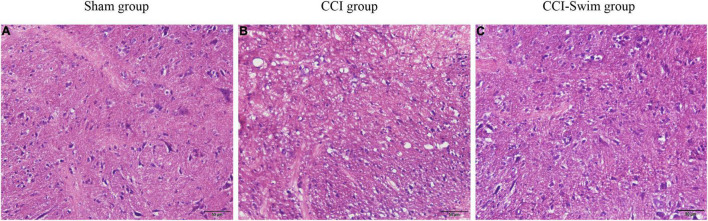
Histological examination of spinal dorsal horn. The hematoxylin-eosin (HE) staining of spinal dorsal horn tissue were observed under light microscopy (×200, bar = 50 μm). Sham group **(A)**, CCI group **(B)**, and CCI-Swim group **(C)**.

The HE staining results showed that the spinal dorsal horn neurons in the Sham group were intact, regular, and orderly and that the nucleolus *in vivo* was distinct. In the CCI group, the spinal dorsal horn neurons were damaged and denatured, with pyknosis, fragmentation, and dissolution of the nucleus, as well as loose surrounding tissues. These features indicate that the CCI model had been successfully prepared. Compared with the CCI group, the CCI-Swim group showed a slightly irregular arrangement of sensory neurons in the spinal dorsal horn and relatively dense surrounding tissues. In general, the histological features of the spinal cord in this group were better than those of the CCI group overall (Figure 3).

### Differentially Expressed Gene Expression in the Spinal Dorsal Horn

**FIGURE 4:**
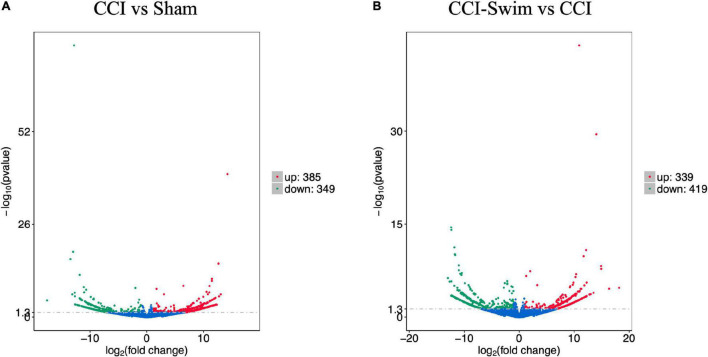
Volcano plots of RNA-sequencing data exhibited the differentially expressed lncRNAs in the spinal dorsal horn between rats in the comparisons of CCI group vs Sham group **(A)**, and CCI-Swim group vs CCI group **(B)**. The red part is significantly upregulated lncRNAs, the green part is significantly downregulated lncRNAs, and the blue part is not significantly expressed lncRNAs.

**FIGURE 5:**
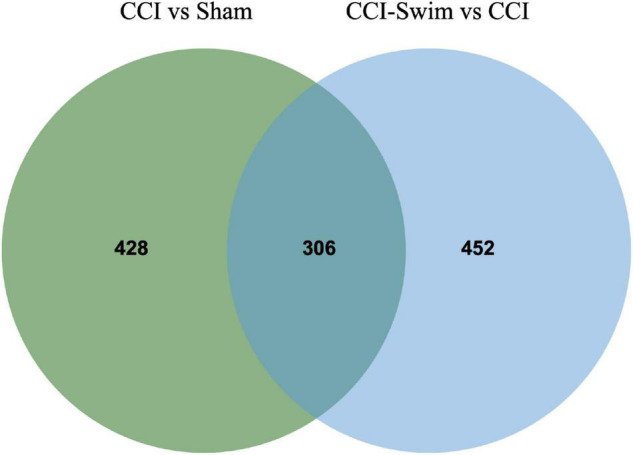
Venn diagram of lncRNAs. The figure shows the intersection of differential expressed lncRNAs between the CCI vs Sham group and the CCI-Swim vs CCI group.

**FIGURE 6:**
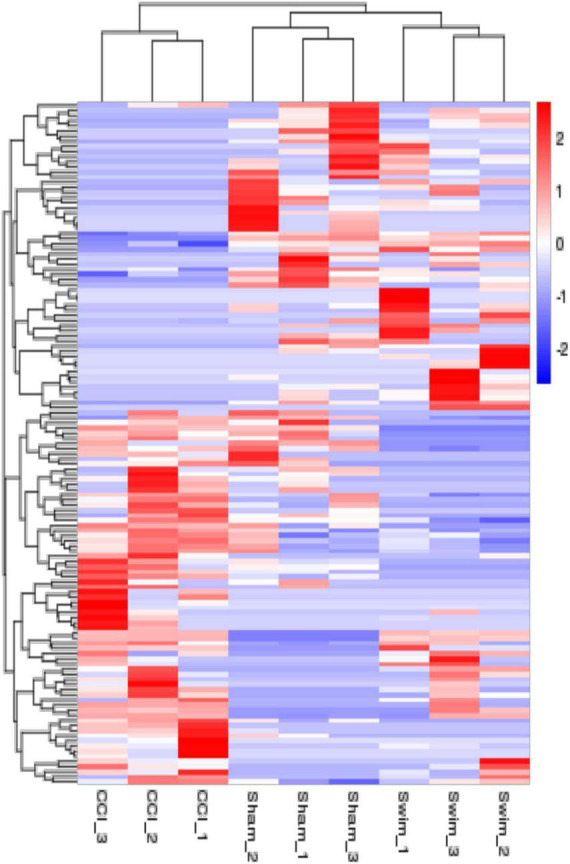
The hierarchical cluster analysis diagram shows the clustering patterns of the Sham, CCI, and CCI-Swim groups of differentially expressed lncRNAs. The red part represents the upregulated lncRNAs and the blue part represents the downregulated lncRNAs.

The quality of the RNA-seq results was evaluated. Comparison of the Sham and CCI groups (Figure 4A) revealed a total of 734 DE lncRNAs, among which 385 lncRNAs were upregulated and 349 were downregulated. The top 20 DE lncRNAs in the Sham vs CCI group are shown in Table 2. There were 758 DE lncRNAs were noted in the CCI-Swim vs CCI group (Figure 4B) and the top 20 DE are listed in Table 3. The Venn diagram indicated 306 intersecting lncRNAs in the two comparisons (Figure 5). Hierarchical cluster analysis revealed the clustering patterns of the three groups of DE lncRNAs (Figure 6).

**TABLE 2:** The top 20 DE lncRNAs in CCI group vs Sham group.

Gene name	Log_2_FC	*P*-value	Regulation
AABR07015080.2	−12.81	<0.001	Down
XLOC_097393	14.21	<0.001	Up
XLOC_309240	−13.00	<0.001	Down
XLOC_176078	−13.45	<0.001	Down
XLOC_314732	12.62	<0.001	Up
XLOC_085264	−11.83	<0.001	Down
XLOC_234469	11.47	<0.001	Up
XLOC_173719	11.46	<0.001	Up
1700066B19Rik	6.44	<0.001	Up
XLOC_189377	11.04	<0.001	Up
XLOC_239191	−11.10	<0.001	Down
XLOC_274480	−2.01	<0.001	Down
XLOC_002354	1.71	<0.001	Up
XLOC_011654	−10.70	<0.001	Down
XLOC_122558	10.52	<0.001	Up
XLOC_305707	−11.04	<0.001	Down
LOC102546683	11.01	<0.001	Up
AABR07053749.1	−12.70	<0.001	Down
XLOC_244676	10.74	<0.001	Down
AABR07058158.1	3.02	<0.001	Up

*DE, differentially expressed; FC, fold change.*

**TABLE 3:** The top 20 DE lncRNAs in CCI-Swim group vs CCI group.

Gene name	Log_2_FC	*P*-value	Regulation
AABR07015080.2	10.90	<0.001	Up
XLOC_309240	14.03	<0.001	Up
LOC108348298	−12.39	<0.001	Down
XLOC_025354	−12.35	<0.001	Down
AABR07067076.1	−11.79	<0.001	Down
XLOC_312531	12.16	<0.001	Up
XLOC_032459	−11.70	<0.001	Down
XLOC_033171	−11.65	<0.001	Down
XLOC_096101	11.72	<0.001	Up
XLOC_189377	−10.99	<0.001	Down
AABR07062823.1	14.90	<0.001	Up
XLOC_243724	14.93	<0.001	Up
XLOC_212511	−10.98	<0.001	Down
XLOC_274480	2.01	<0.001	Up
XLOC_044460	−10.43	<0.001	Down
XLOC_097273	−10.72	<0.001	Down
XLOC_244676	10.36	<0.001	Up
AABR07071299.1	−10.51	<0.001	Down
AABR07015078.1	1.27	<0.001	Up
XLOC_119427	10.25	<0.001	Up

*DE, differentially expressed; FC, fold change.*

**FIGURE 7:**
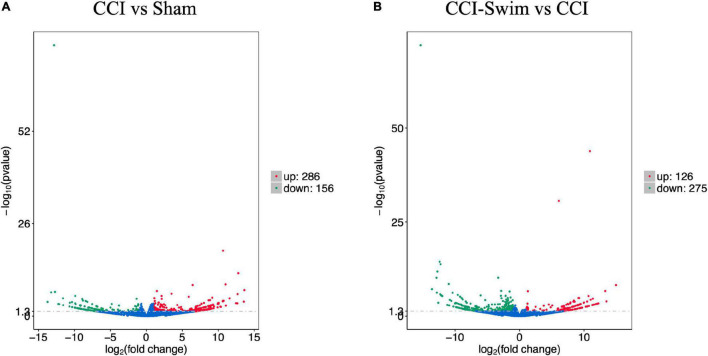
Volcano plots of RNA-sequencing data exhibited the differentially expressed mRNAs in the spinal dorsal horn between rats in the comparisons of CCI group vs Sham group **(A)**, and CCI-Swim group vs CCI group **(B)**. The red part is significantly upregulated lncRNAs, the green part is significantly downregulated lncRNAs, and the blue part is not significantly expressed lncRNAs.

**FIGURE 8:**
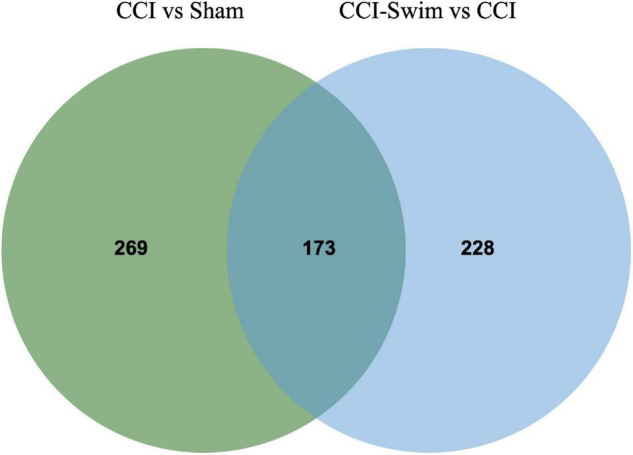
Venn diagram of mRNAs. The figure shows the intersection of differential expressed mRNAs between the CCI vs Sham group and the CCI-Swim vs CCI group.

**FIGURE 9:**
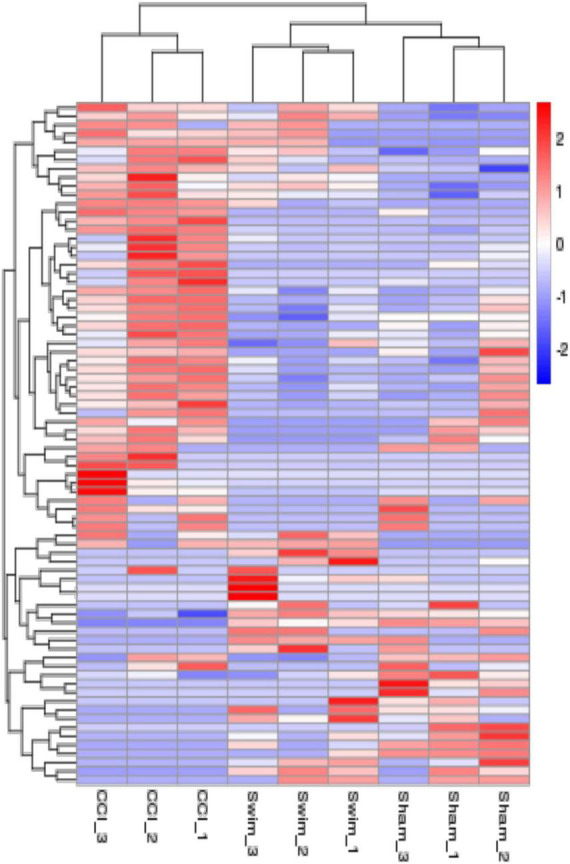
The hierarchical cluster analysis diagram shows the clustering patterns of the Sham, CCI, and CCI-Swim groups of differentially expressed mRNAs. The red part represents the upregulated mRNAs and the blue part represents the downregulated mRNAs.

Figure 7 shows differences in mRNA expression among the three groups. Compared with the Sham group, 442 mRNAs were significantly changed in the CCI group, including 286 upregulated mRNAs and 156 downregulated mRNAs (Figure 7A). The top 20 DE mRNAs in the Sham and CCI groups are listed in Table 4. A total of 401 DE mRNAs were observed in the CCI-Swim and CCI groups (126 upregulated and 275 downregulated; Figure 7B). The top 20 DE mRNAs in the CCI vs CCI-Swim group are detailed in Table 5. The Venn diagram obtained showed 173 intersecting mRNAs, which are listed in Figure 8. Hierarchical clustering analysis of the three groups of DE mRNAs revealed their clustering patterns (Figure 9).

**TABLE 4:** The top 20 DE mRNAs in CCI group vs Sham group.

Gene name	Log_2_FC	*P*-value	Regulation
AABR07015080.2	−12.81	<0.001	Down
LOC100910708	10.66	<0.001	Up
Trdn	12.79	<0.001	Up
LOC685716	11.02	<0.001	Up
1700066B19Rik	6.44	<0.001	Up
LOC100910990	13.64	<0.001	Up
C3	1.47	<0.001	Up
AABR07053749.1	−12.70	<0.001	Down
Senp1	−13.23	<0.001	Down
LOC100910270	3.51	<0.001	Up
LOC100910021	12.69	<0.001	Up
AABR07051731.1	−9.88	<0.001	Down
Clrn3	2.12	<0.001	Up
AABR07043288.1	5.90	<0.001	Up
LOC685699	9.17	<0.001	Up
Adgre1	1.13	<0.001	Up
AABR07017658.1	−11.57	<0.001	Down
Themis	11.57	<0.001	Up
Sgk1	−1.34	<0.001	Down
Vgf	2.15	<0.001	Up

*DE, differentially expressed; FC, fold change.*

**TABLE 5:** The top 20 DE mRNAs in CCI-Swim group vs CCI group.

Gene name	Log_2_FC	*P*-value	Regulation
LOC100910882	−15.35	< 0.001	down
AABR07015080.2	10.90	<0.001	Up
Fam111a	6.08	<0.001	Up
LOC108348298	−12.39	<0.001	Down
Tex11	−12.25	<0.001	Down
Trdn	−12.74	<0.001	Down
Myo3b	−3.32	<0.001	Down
Rgs13	−12.87	<0.001	Down
LOC685716	−10.97	<0.001	Down
LOC100910143	14.94	<0.001	Up
LOC100910990	−13.59	<0.001	Down
Ak7	−2.84	<0.001	Down
Senp1	13.26	<0.001	Up
AABR07015078.1	1.27	<0.001	Up
Pik3c2g	−1.56	<0.001	Down
LOC103690114	−12.83	<0.001	Down
AABR07052585.1	−10.39	<0.001	Down
Nhp2	−12.31	<0.001	Down
Noxred1	−9.58	<0.001	Down
AABR07042937.1	−12.16	<0.001	Down

*DE, differentially expressed; FC, fold change.*

### Gene Ontology Functional Analysis

Gene Ontology analysis was conducted on the target genes with significant changes in DE lncRNAs. Between the CCI and Sham groups, the target genes were enriched in biological process (BP) terms such as muscle structure development, positive regulation of BP, and muscle organ development (*P* < 0.05). The cellular component (CC) terms were significantly related to Z disc, I band, and sarcomere (*P* < 0.05). Molecular functions (MFs) were enriched in terms such as acid phosphatase activity, structural constituent of muscle, and protein binding (*P* < 0.05). The target genes of DE lncRNAs in the CCI-Swim and CCI groups were concentrated in BP terms such as muscle system process, positive regulation of immune system process, and positive regulation of immune response (*P* < 0.05). The most enriched CCs were Z disc, I band, and sarcomere (*P* < 0.05). MF terms were focused on acid phosphatase activity, structural constituent of muscle, and phosphatase activity (*P* < 0.05).

**FIGURE 10:**
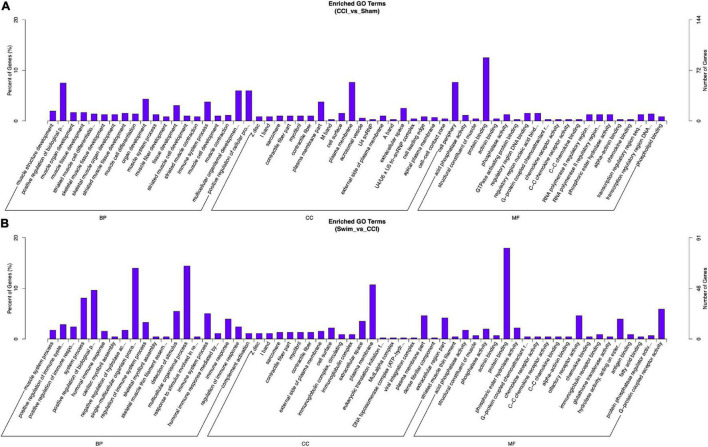
Gene Ontology analysis on target genes of lncRNA. **(A)** CCI group vs Sham group, and **(B)** CCI-Swim group vs CCI group. *N* = 3 per group.

**FIGURE 11:**
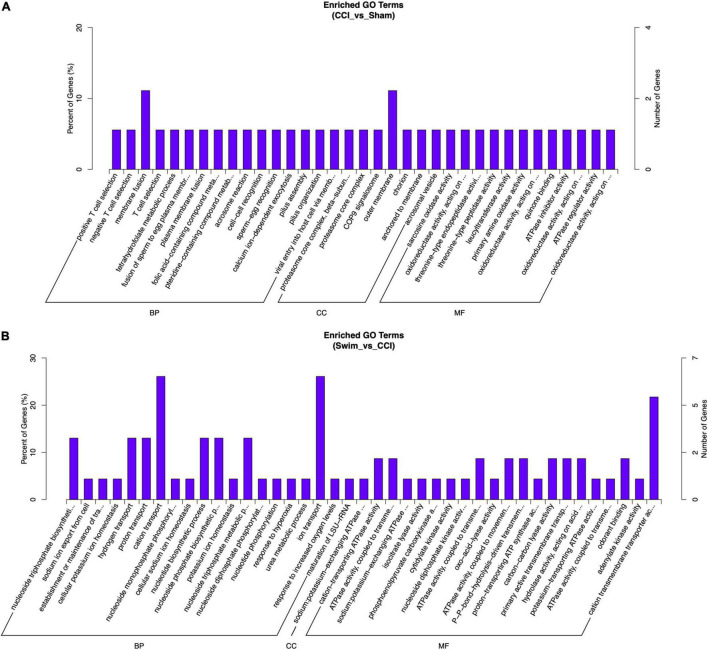
Gene Ontology analysis on differential expression mRNAs. **(A)** CCI group vs Sham group, and **(B)** CCI-Swim group vs CCI group. *N* = 3 per group.

Gene Ontology analysis was also applied to the DE mRNAs of two groups, and the results are shown in Figure 10 (*P* < 0.05). DE mRNAs in the CCI and Sham groups were enriched in BP terms such as positive T cell selection, negative T cell selection, and membrane fusion; DE mRNAs were also enriched in CC terms such as proteasome core complex, beta-subunit complex, proteasome core complex, and COP9 signalosome (*P* < 0.05). The most enriched MF terms were sarcosine oxidase activity, oxidoreductase activity, and threonine-type endopeptidase activity (*P* < 0.05). The DEGs of mRNAs in the CCI vs CCI-Swim group were focused on BPs terms such as nucleoside triphosphate biosynthetic process, sodium ion export from cell, and establishment or maintenance of transmembrane electrochemical gradient (*P* < 0.05). The CC terms were enriched in the sodium:potassium-exchanging ATPase complex. Finally, DE mRNAs were significantly enriched in MF terms such as cation-transporting ATPase activity, ATPase activity, coupled to transmembrane movement of ions, and sodium:potassium-exchanging ATPase activity (*P* < 0.05; Figure 11).

### KEGG Pathway Enrichment Analysis

**FIGURE 12:**
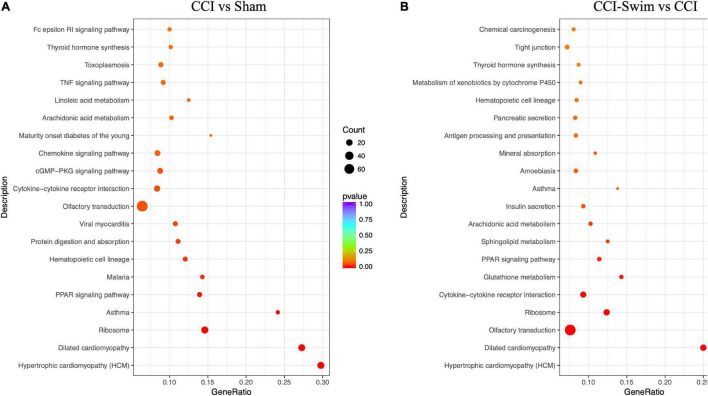
KEGG pathways of the target genes of lncRNAs. **(A)** CCI group vs Sham group, and **(B)** CCI-Swim group vs CCI group. *N* = 3 per group.

**FIGURE 13:**
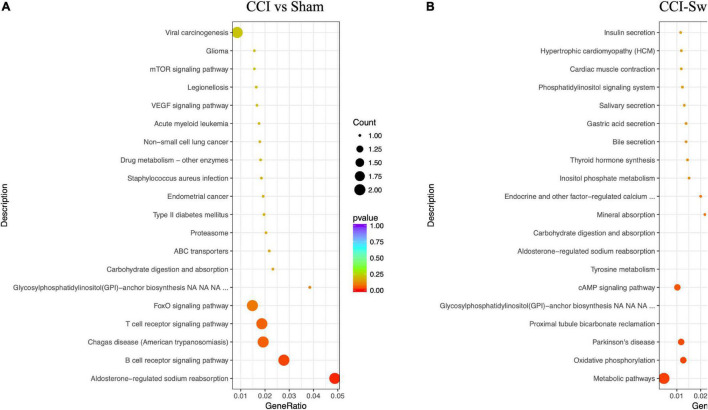
KEGG pathways of the differential expression mRNAs. **(A)** CCI group vs Sham group, and **(B)** CCI-Swim group vs CCI group. *N* = 3 per group.

Figure 12 shows the KEGG pathways of the target genes of DE lncRNAs in the CCI vs Sham groups. The top five KEGG pathways were hypertrophic cardiomyopathy (HCM), dilated cardiomyopathy, ribosome, asthma, and PPAR signaling pathway (*P* < 0.05). In the CCI-Swim vs CCI groups, the top five enriched KEGG pathways were HCM, dilated cardiomyopathy, olfactory transduction, ribosome, and cytokine–cytokine receptor interaction (*P* < 0.05). For mRNAs, DEGs in the CCI and Sham groups focused on aldosterone-regulated sodium reabsorption and B cell receptor signaling pathway. DE mRNAs in the CCI-Swim and CCI groups were significantly enriched in the KEGG pathways of metabolic pathways, oxidative phosphorylation, Parkinson’s disease, proximal tubule bicarbonate reclamation, glycosylphosphatidylinositol–anchor biosynthesis, and cAMP signaling pathway (*P* < 0.05; Figure 13).

### Quantitative Real-Time PCR Analysis

**FIGURE 14:**
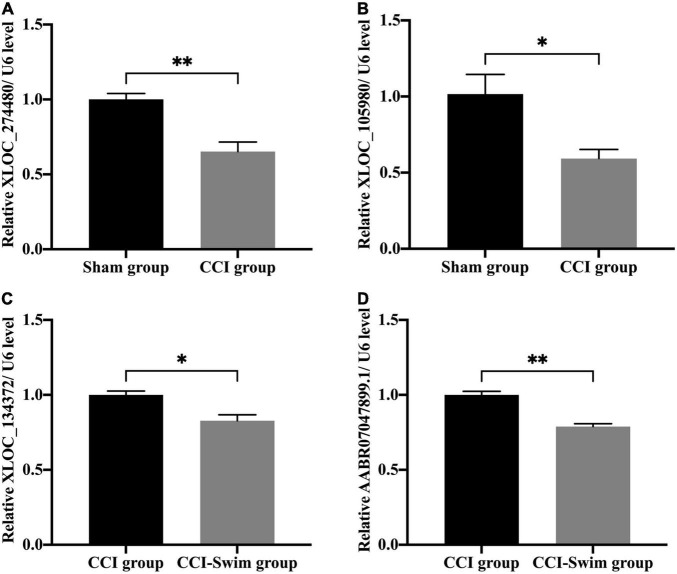
Quantitative real-time PCR validation. The expression levels of lncRNAs **(A)** XLOC_274480, **(B)** XLOC_105980, **(C)** XLOC_134372, and **(D)** AABR07047899.1 in the spinal cord of CCI rats at 28 days post operation. Data was analyzed by independent-samples *t*-test. Values are denoted by mean ± SEM, *N* = 3 per group. *p < 0.05, ^**^p < 0.01.

**FIGURE 15:**
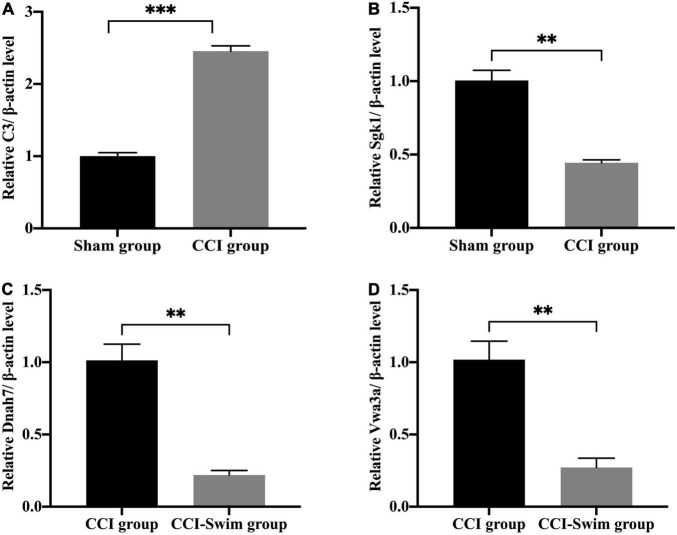
Quantitative real-time PCR validation. The expression levels of mRNAs **(A)** C3, **(B)** Sgk1, **(C)** Dnah7, and **(D)** Vwa3a in the spinal cord of CCI rats at 28 days post operation. Data was analyzed by independent-samples *t*-test. Values are denoted by mean ± SEM, *N* = 3 per group. ^**^p < 0.01, ^***^p < 0.001.

We verified four lncRNAs (i.e., XLOC_274480, XLOC_105980, XLOC_137372, and AABR07047899.1) and four mRNAs (i.e., C3, Sgk1, Dnah7, and Vwa3a) to demonstrate the accuracy of the RNA-seq results. According to Figures 14, 15, the expression changes of the eight DEGs were consistent with the RNA-seq result.

## Discussion

In this study, we found remarkable differences in the expression of lncRNAs and mRNAs in the spinal dorsal horn of rats in the Sham, CCI, and CCI-Swim groups. We also predicted the potential functions of these DEGs by GO and KEGG pathway analyses. The findings are helpful in further explorations of the potential therapeutic targets of NP.

Previous studies demonstrated that the potential mechanisms of NP include abnormal heterotopic activity of the injurious nerve, peripheral and central sensitization, impaired inhibitory regulation, and pathological activation of microglia (Meacham et al., 2017; Inoue and Tsuda, 2018; Tozaki-Saitoh and Tsuda, 2019; Finnerup et al., 2021). The current treatment methods for NP mainly include pharmacology, non-pharmacology and interventional therapy (Nijs et al., 2015; Macone and Otis, 2018). However, most existing treatments are limited in their effectiveness in controlling pain (Gilron et al., 2015; Xu et al., 2016; Szok et al., 2019). Therefore, exploring new alternative treatment methods for NP remains an urgent necessity.

Given the increasing popularity of exercise in the field of medicine, the treatment of NP by exercise has become a research hotspot (Safakhah et al., 2017; Guo et al., 2019; Ma et al., 2019; Palandi et al., 2020). Kuphal et al. (2007) demonstrated that in NP model mice, swimming for 7 days can significantly increase the pain threshold of thermal hyperalgesia, while swimming for 18–20 days can significantly improve the thermal hyperalgesia and cold allodynia in CCI rats. Farzad et al. (2018) reported that after 4 weeks of swimming training could significantly decrease allodynia and hyperalgesia in rats with NP. The authors thus believed that the improvement of pain tests was associate with GAD65 (Farzad et al., 2018). Another study demonstrate the expressions of inflammatory cytokines IL-4, IL-1RA, and IL-5DE were upregulated in the spinal cord of mice with peripheral nerve injury. Two weeks of treadmill exercise could significantly suppress the expression of inflammatory factors and improve pain behaviors (Bobinski et al., 2018). The results of our own research presented that the mechanical pain threshold of CCI rats significantly increased on day 21 and day 28 after swimming training. These studies have confirmed that exercise has a positive effect on NP.

Previous research showed that lncRNAs participated in the processes of NP and regulate NP-related gene expression (Li et al., 2019; Wu et al., 2019). Zhou et al. (2017a) utilized the second-generation sequencing method to observe the expressions of lncRNA and mRNA in the spinal cord of SNI rats at different time points. The authors’ results showed that lncRNAs and mRNAs in rats changed significantly at each measuring time point measured. The authors then explored the profiles of DEGs in the spinal cord of NP rats through GO and KEGG analyses (Zhou et al., 2017a). Another article employed microarray analysis to show the level of DEGs in the spinal cord of spinal nerve ligation (SNL) rats. A total of 511 DEGs of lncRNAs and 493 DEGs of mRNAs were observed on the 10th day after SNL surgery. Functional analysis indicated that the DEGs most enriched in SNL included immune response, defense response, and inflammatory response, thus revealing the potential mechanisms in NP (Jiang et al., 2015).

Some existing studies have explored DEGs in NP through gene sequencing and gene functional analysis. However, there was few literatures analyzing the potential mechanisms of the improvement of NP through exercise. Hence, we explored DE lncRNAs and mRNAs by means of second-generation sequencing and conducted further functional analysis. Among the two comparison groups, we revealed 173 intersecting mRNAs. In these intersecting mRNAs, Dnah6 was significantly upregulated in the CCI vs Sham group but downregulated in the CCI-Swim vs CCI group. Similarly, Zhou et al. (2017a) demonstrated that the expression of Dnah6 is significantly increased in the NP model rats, which is consistent with our results. The mRNAs Pkd1l2, C3, Adgre1, and Plac9 have showed in previous RNA-seq literatures related with NP, and their significant alterations are consistent with our results (Zhou et al., 2017a,b; Du et al., 2018). Changes in the DE of these mRNAs were consistent with our sequencing results, which means they could be potential target genes for the development of NP. In addition, we found that some significantly altered mRNA such as C3, Sgk1, and VGF were associated with NP. Studies have revealed that C3 levels are positively correlated with the degree of neurological impairment in a variety of neurodegenerative diseases (Aeinehband et al., 2015). Sgk1 is genetically regulated by cellular stress and several hormones. It can activate a variety of ion channels and participate in BPs (Arteaga et al., 2008; Lang and Shumilina, 2013). VGF is selectively expressed in neuroendocrine cells and neurons, including central and peripheral nervous systems (Ferri et al., 2011). These mRNAs may be biomarkers for the treatment of NP, and future studies can focus on these mRNAs for further research.

The results of GO analysis of DE lncRNA target genes revealed the GO terms, such as extracellular space, plasma membrane, phosphatase activity, and protein binding, consistent with previous NP related studies (Zhou et al., 2017a,b; Du et al., 2018; Mao et al., 2018; Wang et al., 2019; Li et al., 2021). These GO terms were significantly expressed among the two group comparisons, thus suggesting that they may be the potential pathways of the effects of exercise on NP.

In the CCI vs Sham group and the CCI-Swim vs CCI group, the KEGG pathway enriched by lncRNA target genes showed significant changes in cytokine–cytokine receptor interaction, Asthma, Ribosome, and PPAR signaling pathway. The enrichment degree of these pathways in the CCI vs Sham group was significantly changed, which was in accordance with existing researches (Zhou et al., 2017a; Du et al., 2018; Wang et al., 2019; Li et al., 2021). In addition, these enrichment pathways changed significantly again after swimming in the CCI-Swim group, suggesting that they may be a potential pathway for exercise to improve NP.

In clinical work, medical workers may recommend appropriate exercise for NP patients to improve the symptoms of pain, which has guiding significance for the clinical treatment of NP. Although we investigated the underlying mechanism of exercise therapy for NP by sequencing, there are still some limitations in our study. For example, the results showed significant improvements in the pain behavior of rats in the CCI-Swim vs CCI groups on days 21 and 28 postoperatively. However, RNA-seq was only performed on the 28th day after CCI surgery; thus, future studies should also conduct RNA-seq on the 21st day to explore more NP-related DEGs. Moreover, whereas swimming is usually a voluntary exercise in humans, it is a forced exercise in rats. Therefore, future studies on the role of exercise in improving NP may include forms of exercises considered voluntary in animals, such as autonomous wheel exercise.

## Conclusion

In this study, we demonstrated that swimming exercise can relieve NP. RNA-seq analysis was utilized to identify the DEGs of lncRNAs and mRNAs among Sham, CCI, and CCI-Swim groups. The DEGs obtained may be potential treatment targets for NP. We also conducted bioinformatics analysis of dysregulated lncRNAs and mRNAs and obtained information that could lead to a better understanding of the mechanism of exercise in improving NP.

## Data Availability Statement

The raw data have been uploaded to the Sequence Read Archive (SRA) database of NCBI under accession number PRJNA768994.

## Ethics Statement

The animal study was reviewed and approved by the Ethics Committee of Scientific Research of Shanghai University of Sport.

## Author Contributions

X-QW, QX, and GS contributed to conception and design of the study. GS, J-BG, and Y-LZ organized the related experiments. ZY, XS, and Y-MC performed the statistical analysis. GS and X-QW wrote the first draft of the manuscript. QX, J-BG, W-MZ, Y-ZW, and Y-LZ wrote sections of the manuscript. All authors contributed to manuscript revision, read, and approved the submitted version.

## Conflict of Interest

The authors declare that the research was conducted in the absence of any commercial or financial relationships that could be construed as a potential conflict of interest.

## Publisher’s Note

All claims expressed in this article are solely those of the authors and do not necessarily represent those of their affiliated organizations, or those of the publisher, the editors and the reviewers. Any product that may be evaluated in this article, or claim that may be made by its manufacturer, is not guaranteed or endorsed by the publisher.

## Footnotes

1
http://www.genome.jp/kegg/
