# Supplementary material for: Long Non-coding RNA and mRNA Expression Change in Spinal Dorsal Horn After Exercise in Neuropathic Pain Rats
Source: Front Mol Neurosci. 2022 Mar 30;15:865310. doi: 10.3389/fnmol.2022.865310 (PMC9005956; doi:10.3389/fnmol.2022.865310)
Supplement: Supplementary file 1 [file Table_1.DOCX]

Table S1. Sequencing data quality summary

| Sample_name | Raw_reads | Clean_reads | Raw_bases(G) | Clean_bases(G) | Error rate(%) | Q20(%) | Q30(%) | GC_content(%) |
| --- | --- | --- | --- | --- | --- | --- | --- | --- |
| CCI_1 | 115450150 | 112739894 | 17.32 | 16.91 | 0.03 | 97.27 | 92.46 | 46.32 |
| CCI_2 | 102558912 | 100651970 | 15.38 | 15.1 | 0.03 | 97.38 | 92.63 | 46.32 |
| CCI_3 | 84511776 | 82887138 | 12.68 | 12.43 | 0.03 | 97.68 | 93.3 | 46.76 |
| Sham_1 | 108948258 | 107041958 | 16.34 | 16.06 | 0.03 | 97.5 | 92.89 | 46.96 |
| Sham_2 | 105121278 | 103187690 | 15.77 | 15.48 | 0.03 | 97.82 | 93.61 | 46.36 |
| Sham_3 | 110522094 | 108459040 | 16.58 | 16.27 | 0.03 | 97.49 | 92.89 | 47 |
| Swim_1 | 95543536 | 93109152 | 14.33 | 13.97 | 0.03 | 97.19 | 92.32 | 47.26 |
| Swim_2 | 96665026 | 94995658 | 14.5 | 14.25 | 0.03 | 97.43 | 92.77 | 47.09 |
| Swim_3 | 111969422 | 109618546 | 16.8 | 16.44 | 0.03 | 97.25 | 92.47 | 46.71 |
